# Does information from ClinicalTrials.gov increase transparency and reduce bias? Results from a five-report case series

**DOI:** 10.1186/s13643-018-0726-5

**Published:** 2018-04-16

**Authors:** Gaelen P. Adam, Stacey Springs, Thomas Trikalinos, John W. Williams, Jennifer L. Eaton, Megan Von Isenburg, Jennifer M. Gierisch, Lisa M. Wilson, Karen A. Robinson, Meera Viswanathan, Jennifer Cook Middleton, Valerie L. Forman-Hoffman, Elise Berliner, Robert M. Kaplan

**Affiliations:** 1 Brown University Evidence-based Practice Center, Providence, USA; 2 Duke University Evidence-based Practice Center, Durham, USA; 3 Johns Hopkins University Evidence-based Practice Center, Baltimore, USA; 4 RTI/UNC Evidence-based Practice Center, Chapel Hill, USA; 5 Agency for Healthcare Research and Quality, Rockville, USA; 6 Stanford University Clinical Excellence Research Center, Stanford, USA

## Abstract

**Background:**

We investigated whether information in ClinicalTrials.gov would impact the conclusions of five ongoing systematic reviews.

**Method:**

We considered five reviews that included 495 studies total. Each review team conducted a search of ClinicalTrials.gov up to the date of the review’s last literature search, screened the records using the review’s eligibility criteria, extracted information, and assessed risk of bias and applicability. Each team then evaluated the impact of the evidence found in ClinicalTrials.gov on the conclusions in the review.

**Results:**

Across the five reviews, the number of studies that had both a registry record and a publication varied widely, from none in one review to 43% of all studies identified in another. Among the studies with both a record and publication, there was also wide variability in the match between published outcomes and those listed in ClinicalTrials.gov. Of the 173 total ClinicalTrials.gov records identified across the five projects, between 11 and 43% did not have an associated publication. In the 14% of records that contained results, the new data provided in the ClinicalTrials.gov records did not change the results or conclusions of the reviews. Finally, a large number of published studies were not registered in ClinicalTrials.gov, but many of these were published before ClinicalTrials.gov’s inception date of 2000.

**Conclusion:**

Improved prospective registration of trials and consistent reporting of results in ClinicalTrials.gov would help make ClinicalTrials.gov records more useful in finding unpublished information and identifying potential biases. In addition, consistent indexing in databases, such as MEDLINE, would allow for better matching of records and publications, leading to increased utility of these searches for systematic review projects.

**Electronic supplementary material:**

The online version of this article (10.1186/s13643-018-0726-5) contains supplementary material, which is available to authorized users.

## Background

Information biases, including publication bias, time-lag bias, selective outcome reporting bias, and selective analysis bias, are major threats to the validity and usability of systematic reviews. Systematic reviewers have pursued two method approaches for dealing with information bias. One is to detect (and possibly correct results for) information bias based only on the identified studies (e.g., using funnel-plot-based methods [1–4] or sensitivity analyses to account for possible missing data [5–7] or comparing outcomes listed under Methods versus those reported under Results in published manuscripts [8]). A second is to examine trial registries, survey researchers, and peruse the gray literature to identify unpublished study results or ongoing studies.

Clinical trial registries that include prospective registration of study protocols, as well as in some cases summarized results (e.g., the National Library of Medicine ClinicalTrials.gov registry and registry networks, such as International Clinical Trials Registry Platform [ICTRP]), are a good source of this information. Regulations established in 1997 and expanded under the Food and Drug Administration Amendments Act of 2007 [9, 10], National Institutes of Health policy, and International Committee of Medical Journal Editors (ICMJE) guidance [11] have motivated industry sponsors and academic researchers to prospectively register their studies. This collection of policies includes requirements for registering studies including documenting type of study, intervention, trial phase, funding source, and outcomes, and types of data to be included within a study record. Enforcement of these policies has evolved since the launch of ClinicalTrials.gov in 2000. Clarifications on what information should be included in ClinicalTrials.gov have been issued to the research community to ensure compliance and timely submission of appropriate data to the registry [12]. Advocates of clinical trial registration emphasize the role of registry platforms to disseminate aggregated results to researchers, clinicians, and study participants. Registries enhance transparency by providing an inventory of studies that are in progress or have been completed [13, 14].

Empirical analyses of prospective registration of studies (defined here as registration of investigational studies prior to enrollment of the first patient or, for observational studies, prior to initial analyses) can inform on the time between study completion and publication, the number of unpublished studies, the fidelity of studies to registered protocols, and the congruence of study results between registry records and publications [15–18]. The current Agency for Healthcare Research and Quality Evidence-based Practice Center (EPC) Program guidance recommends searching trial registries [19], but it is labor-intensive and the utility of the additional effort required is uncertain.

The objective of this project was to assess whether information from ClinicalTrials.gov would affect the conclusions of five ongoing systematic reviews.

## Methods

To assess the impact of information derived from ClincialTrials.gov on systematic reviews, five ongoing systematic reviews were selected. Our goal was to measure prospectively the resource use and payoff of ClinicalTrials.gov searches in real-time across a variety of topics. The full method sections for each project are reproduced in Additional file 1.

### Selection of reviews

The Agency for Healthcare Research and Quality sent a request for proposals to members of their network of evidence-based practice centers for analysis of the evaluation of ClinicalTrials.gov data in ongoing reports. From the submitted proposals, they chose to fund five projects that were based in different clinical areas and populations and spanned different types of interventions, in order to evaluate the impact across as broad a range as possible. The selected reviews include as follows: Prevention and treatment of diabetic peripheral neuropathy [20], Evaluating treatment for infertility [21], Omega-3 fatty acids and cardiovascular disease [22], tympanostomy tubes in children with otitis media [23], Strategies to improve mental health care for children and adolescents [24].

### Definition of terms

For the purposes of this project, a *study* is the actual research conducted, a *record* is the ClinicalTrials.gov report of the study, and a *publication* is a journal article report of the study. Each of the five case studies is referred to as a *project*. The original systematic review on which each project is based is the *report*.

### General methods followed by all teams

These five projects were conducted by four separate EPCs to ensure consistency in searching and screening between the systematic reviews and the report [25–29]. Details are given in Table 1. In general, they included the following: A search of ClinicalTrials.gov at the date of the original review’s update search, screening the records using the original review’s eligibility criteria, extracting information from the records, and assessing risk of bias and applicability. Relevant studies identified in registry records were compared to those included in the review.

**Table 1:** Methods employed across projects assessing utility of ClinicalTrials.gov searches in ongoing systematic reviews

Protocol element	Diabetic peripheral neuropathy	Infertility	Omega-3 and CVD	Mental health	Tympanostomy tubes
Search ClinicalTrials.gov using terms adapted from the systematic review	x	x	x Also searched ICTRP	x	x Also searched ICTRP
Screened records using same criteria as systematic review	x	x	x	x	x
Data Extraction from ClinicalTrials.gov	x	x	x	x	x
Compared records to publications	x	x	x	x Also emailed authors	x
Population	x	x	x	x	x
Intervention	x	x	x	x	x
Outcome definition	x	x	x	x	x
Population baselines	x	x	x	x	x
Results	x	x	x	x	x
Risk of bias			x		x
When there was only a ClinicalTrials.gov record					
Recruitment status (as listed in ClinicalTrials.gov)	x	x	x	x	x
Dates* (study start and completion dates)	x	x	x	x	x
Results reported in ClinicalTrials.gov	x	x	x	No studies with results reported only in records	x
Re-analyzed ongoing review with new results	x	No records had usable results	No records had usable results	The systematic review did not have quantitative analyses	No records had usable results
Country (site information in ClinicalTrials.gov)			x		x

*Extracted dates from ClincialTrials.gov records to establish how many are more than 3 years old

Studies were matched to publications using the citations listed in the “More Information” section of the Study Details tab of the ClinicalTrials.gov record or the National Clinical Trial (NCT) number given in the MEDLINE® record. Where no match could be identified using the NCT identifier, the teams manually searched MEDLINE using terms for the interventions and principal investigator as search criteria.

Each team abstracted selected variables from the ClincalTrials.gov records to determine whether key study design variables and reported outcomes matched information in the published manuscript. Variables abstracted matched those extracted from reports for the systematic reviews and included date of completion, number of study arms and N of the study, intervention description, study design, outcomes measures, analysis approach and subgroup analyses, numerical results, and risk of bias. Each team then evaluated the impact of the evidence found in the registries on the conclusions and strength of evidence in the report in different ways. The project protocols were prospectively published on the AHRQ Web site (https://effectivehealthcare.ahrq.gov/topics/transparency-reporting/overview/).

### Important method variations between projects

Variations between projects include the following: Two projects also searched the World Health Organization’s (WHO) International Clinical Trials Registry Platform (ICTRP) [25, 26]. This registry does not include results, but the team was able to compare other study details for these records. These results are included in the reported results for these two projects. A second team contacted the authors of included studies (via phone and email) to collect information about rationale for use or non-use of ClinicalTrials.gov or other archive sites for information on study conduct and processes [27]. In addition, four of the five projects included a plan to incorporate new results in meta-analyses where feasible [25, 26, 28, 29].

### Assessing the impact of records on the review

In assessing the impact on the systematic review of the records found, the teams looked at three categories of studies. The first category included studies that had both a ClinicalTrials.gov record and a publication. For these studies, each team compared the record to the publication, in terms of design details, population description, sample size, details about the interventions, outcomes, and results if they were given. If results were identified in the record that were not found in the publication, these were incorporated into the review as applicable. The second category included studies that had a record in ClinicalTrials.gov but no publication. For these studies, the team documented the study status (whether it was completed, discontinued, or ongoing) and relevant dates. If the record reported results, these were extracted using the forms used for the original review and incorporated into the systematic review if they were applicable and usable. The final category included studies with a publication but no record. These studies were evaluated for potential reasons why they were not included in the registry.

## Results

As Table 2 shows, the proportion of ClinicalTrials.gov (and ICTRP) records that could be matched to publications ranged from a low of 3% of all studies identified in either the literature or ClicinalTrials.gov search for the tympanostomy tubes review to 43% in the infertility review. Between 11 and 30% of records did not have an associated publication. Of the records without associated publications, the number of records that were more than 3 years old varied widely. The cutoff of 3 years was chosen because it indicated that there had been sufficient time for them to go through the publication process. Finally, in most cases, most of the publications in the systematic reviews were not registered in ClinicalTrials.gov. Of these, most were conducted and completed prior to ClinicalTrials.gov’s inception date or were not conducted in the USA. Despite the fact that four of the five projects intended to rerun meta-analyses with new results to measure changes in magnitude and direction of effect, only one project was able to find new study-level results that would allow them to do so. These results are presented in the section “Studies that had a ClinicalTrials.gov record but no publication” below.

**Table 2:** Numbers of ClinicalTrials.gov records and publications found

	Diabetic peripheral neuropathy	Infertility	Omega-3 and CVD	Mental health	Tympanostomy tubes
Total studies with ClinicalTrials.gov record or publication	106	28	141	20	200
Studies with both ClinicalTrials.gov record and publication All (%)	30 (28)	12 (43)	26 (18)	4 (20)	6 (3)
ClinicalTrials.gov record only					
All (%)	23 (22)	4 (14)	43 (30)	3 (15)	22 (11)
With results/all	• 7/23	• 4/4	• 0/43	• 0/3	• 2/22
> 3 years old/all	• 13/23	• 2/4	• 9/43	• 0/3	• 5/22
Total studies with ClinicalTrials.gov record or publication					
All (%)	53(50)	12 (43)	72 (52)	13 (65)	172 (86)
Since 2000/all	• 30/53	• 0/12	• 58/72	• 13/13	• 97/172

### Studies that had both a record and publication

We found relatively good agreement between the registration records and publications, where we found both, especially when it came to the design elements and intervention descriptions, as well as in specific results where they were included in the record. We did find some discrepancies in lists of outcomes between how the outcomes were pre-specified in the record and reported in the publication (shown in Table 3). For example, in the omega-3 project, the team found that of all the outcomes, 71 (a majority) were recorded in both ClinicalTrials.gov and the publications; 2 (both clinical outcomes) were reported in only ClinicalTrials.gov records and not included in the publications; and 25 appeared for the first time in the publications. In this report, these were primarily intermediate outcomes (e.g., lipids and blood pressure), though in three papers they were clinical outcomes, including nonfatal stroke, myocardial infarction, and revascularization. The direction, magnitude, and statistical significance of study results for specific outcomes were similar whether they were pre-specified in ClinicalTrials.gov or not (e.g., omega 3 groups had significantly lower triglycerides in all papers, regardless of whether triglycerides were a pre-specified outcome in the record or not). In terms of primary and secondary outcomes, we found that, while it is easy to identify the primary outcome in the record, it is more challenging in the papers, because it is seldom specified and different papers from the same study may report different outcomes as though they were primary. Thus, we were not able to draw any conclusions about type of outcome.

**Table 3:** Outcomes reported in records versus publications

	Diabetic peripheral neuropathy	Infertility	Omega-3 and CVD	Mental health	Tympanostomy tubes
Outcomes reported in both ClincalTrials.gov record and Publication n/N (percent)	18/45 (40)	11/20 (55)	71/98 (72)	0/7 (0)	24/35 (69)
Outcomes reported in ClinicalTrials.gov record only (percent)	5/45 (11)*	3/20 (15)	2/98 (2)	2/7 (29)	8/35 (23)
Outcomes reported in Publication only (percent)	22/45 (49)	6/20 (30)	25/98 (26)	5/7 (71)	3/35 (8)

*One record had results

It is possible that this table is an underestimation of the actual discrepancies between prespecified and reported outcomes, because many of the records were not prospectively registered (defined as registration prior to enrollment of the first patient for RCTs or prior to initial analyses for observational studies). For example, in the tympanostomy tubes project, of the 28 total relevant records, only 15 were prospectively registered, and none of the six records with matching publications was prospectively registered [25].

### Studies that had a ClinicalTrials.gov record but no publication

Between 11 and 30% of all studies had a record but no associated publication (Table 1). However, these numbers may not be totally accurate, because we found it difficult to match records to publications, especially where trials were not indexed to crosswalks between PubMed and ClinicalTrials.gov. The authors of the treatment for infertility project attempted to automate the matching process to streamline finding relevant records but were unable to do so. They planned to use a semi-automated process within the bibliographical database (EndNote® Version X7; Thomson Reuters, Philadelphia, PA), but this approach proved infeasible due to inconsistent assignment of NCT identifiers to EndNote fields [28]. Thus, all matching in all projects was accomplished by manual review, using NCT identifiers in PubMed records, abstracts, or full-text articles or using the “related citations” sections of ClinicalTrials.gov records.

**Fig. 1:**
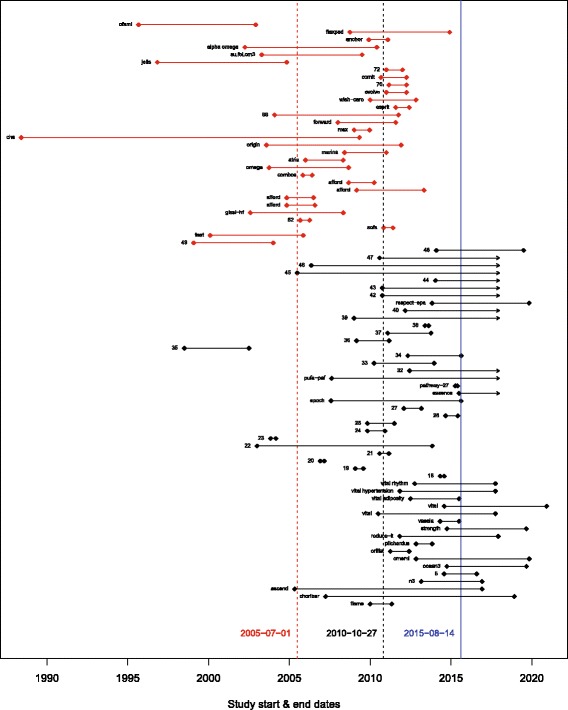
Timing of studies in the omega-3 review. Each line represents a record, with the dots representing the start and end dates as reported in the registry. Red lines indicate studies in with a record and a publication; black lines indicate studies with a record only. The red dashed line is the mean start date for studies with a record and a publication. The black dashed line is the mean start date for studies with a record only. The blue solid line is the date of the ClinicalTrials.gov search. Lines with arrows indicate records that did not give an estimated completion date. In two cases, no start date was given, so the team used the date of entry into the registry

In general, teams found that the records without matching publications were more recent: between 43.5 and 100% were ongoing or had completion dates within the previous 3 years. Figure 1 is from the Omega-3 Fatty Acids and Cardiovascular Disease project. In Fig. 1, each line represents a record, with the dots representing the start and end dates as reported in the registry. Red lines represent studies that were in both the review and the registry; studies represented by black lines did not have a corresponding publication. The mean start date of the studies included in the review (indicated by the red dashed line) was about 5 years earlier than the mean start date of the studies not in the review (indicated by the black dashed line). In addition, many of the studies not in the review were not completed at the time of the search (blue line), which explains why they did not have publications or results [26].

In the review on diabetic peripheral neuropathy, the team found that more than half of the unpublished studies (i.e., with ClinicalTrials.gov records only) were completed more than 3 years earlier (13 of 23, 56.5%), enough time that the results should be published. Seven of these 13 studies reported results in ClinicalTrials.gov (Table 1), suggesting either completion of the study or reporting of interim results. The team also found that, for almost all interventions, the new results had no or minimal effect on the findings of the review. Updated meta-analyses illustrated a pattern of ClinicalTrials.gov records reporting a less-dramatic effect of treatments on pain compared with placebo than the effects reported in the published studies. For both treatments (pregabalin and oxycodone), the overall pattern appeared to be temporal rather than suggestive of publication bias [29]. These results could be a result of time-lag bias or a reflection of the fact that early studies are usually done in the populations most likely to benefit from the intervention.

Interestingly, only one new article was identified through this process. One team found a new publication from a study included in the original review, which had been published since the last update of the review search. The results in this newly identified manuscript were added to the review, but did not change the direction or magnitude of the results for those outcomes [26].

### Studies that had a publication but no registry record

Between 43 and 86% of publications could not be matched to a ClinialTrials.gov record (Table 1). Many of these studies were published before 2000, were not randomized controlled trials, or were not conducted in the USA. Studies conducted outside of the USA that are not published in ICMJE journals may not face the same regulatory and funding requirements to register in ClinicalTrials.gov as those published in the USA or may have been registered in the registry of their home country. For example, in the tympanostomy tubes project, 172 studies, 54 of which were randomized controlled trials, were not found in the registry search. Looking at the randomized controlled trials, 22 were older than ClinicalTrials.gov’s inception date of 2000, and another 13 were not conducted in the USA [25].

The authors of the review on Strategies to improve mental health care for children and adolescents reached out to 15 authors to ask them why they had or had not registered their studies [27]. Mental health in children and adolescents entail by nature complex and system-level interventions, which are not standard in clinical trials registries, though this information could be vital to the next generation of implementation studies on the critical components of their interventions. Ten authors replied, six had not attempted to register their studies and therefore noted no barriers. Of the four that had registered their studies, three noted no barriers to registering, one noted a mismatch between the nature of the study and the purpose of clinicaltrials.gov, and three questioned the utility of registering studies of complex interventions in clinicaltrials.gov.

## Discussion

Overall, our analysis across these five projects lead to the conclusion that performing searches of ClinicalTrials.gov and including available results in analyses had a minimal effect on the results of the systematic review projects; of the five projects, only one found enough evidence to measure whether new information would change the results of meta-analyses, and their conclusion was that the new data did not change the direction or magnitude of the results [29]. Recent work by Baudard and colleagues reviewed existing systematic reviews of pharmaceutical agents. Their analyses also determined that additional data yield from registries did not change in the interpretation of the results [30]. Further, for the five reviews in this project, the records we found were in large part not sufficient to establish or rule out bias, including publication bias, time-lag bias, and selective reporting bias.

While searching registries did not affect the result of the reviews, registry records do have the potential to add value to systematic reviews. These searches revealed studies, outcomes, and results not included in the systematic reviews. For example, two teams found studies with results for which there was no matching publication, and one team was able to add the new results to meta-analyses [29]; the other was not, because the results were not given in a format that could be used [25]. As noted by others, records may contain results for outcomes that are not reported in the publications, such as adverse events [31]. However, at this point, many records do not contain sufficient information to incorporate new results. We found many records in ClinicalTrials.gov that did not have results or publications, and about 30% of these studies were completed more than 3 years ago. Because only 13 of the 29 studies with records only that were completed more than 3 years ago reported results, we could not determine whether the unpublished studies would have impacted the conclusions of our reviews.

Another value of these searches is the opportunity to compare prospective records with their respective publications to assess discrepancies between the data in the review and the registration information in ClinicalTrials.gov to see whether those discrepancies suggest bias [8, 32]. In this project, we found discrepancies difficult to interpret, in part because of the limited information in the ClinicalTrials.gov records and the fact that so many studies are retrospectively registered.

A third value is to document the gaps in evidence and detail what additional studies are needed to address these gaps. Registries can be used to refine research needs, inform whether identified gaps are being addressed, and provide a record of ongoing research. This, in turn can be used in combination with the existing evidence to help determine future research needs.

These potential benefits come at a cost, in terms of staff time (i.e., in manually screening, matching, and analyzing records). In the project on treatment for infertility, the team devoted an estimated 74.5 total hours to the ClinicalTrials.gov searches, screening, and analyzing new evidence [28]. Better and more consistent reporting in the major databases, such as MEDLINE, might improve this and even allow for automation. In a study by Zarin and colleagues in 2011, of the 2324 ClinicalTrials.gov result entries, only 14% were linked to a PubMed citation through the NCT number [33].

### Limitations

The primary limitation of this project is that it was performed by four separate teams, using different protocols for different topic areas. While the protocols were similar in concept, they differed in some specifics. For example, one project did not look at adverse event data [29].

A second limitation is that two of the projects were relatively small, including few studies [27, 28], and the others included a large number of studies that were relatively old, predating ClinicalTrials.gov in many cases and the results requirement in most [25, 26, 29]. It is possible that ClinicalTrials.gov will have greater added utility for newer interventions and larger projects.

A third limitation is that most projects limited their registry searches to ClinicalTrials.gov and may have missed studies registered in other trials. However, the two projects that also searched the WHO ICTRP’s registry found very few new studies, none of which had results [25, 26].

Finally, it is important to note that this article is summarized from a case series of five systematic reviews, albeit on a range of topics, and thus the results should be interpreted with caution and may not be generalizable.

## Conclusion

Searches of ClinicalTrials.gov records did not influence the conclusions of five systematic review projects, and we were unable to draw conclusions about reporting bias due to lack of information in the records. Improved prospective registration of trials and consistent reporting of results in ClinicalTrials.gov would go a long way to make ClinicalTrials.gov records more useful in finding unpublished information and identifying potential biases. In addition, more consistent indexing in databases, such as MEDLINE, and in ClinicalTrials.gov would allow for better cross-matching of records and publications, leading to increased utility of these searches for systematic review projects.

## Additional file

Additional file 1: Methods from Each Project. (DOCX 46 kb)

## Abbreviations

AHRQ: Agency for Healthcare Research and Quality
EPC: Evidence-based Practice Center
ICJME: International Committee of Medical Journal Editors
ICTRP: International Clinical Trials Registry Platform
NCT: National Clinical Trial
RCT: Randomized Controlled Trial
WHO: World Health Organization
