# Supplementary material for: Does information from ClinicalTrials.gov increase transparency and reduce bias? Results from a five-report case series
Source: Syst Rev. 2018 Apr 16;7:59. doi: 10.1186/s13643-018-0726-5 (PMC5902969; doi:10.1186/s13643-018-0726-5)
Supplement: Supplementary file 1 — Methods from Each Project. (DOCX 46 kb) [file 13643_2018_726_MOESM1_ESM.docx]

**Additional file 1: Methods from Each Project**

**Prevention and treatment of Diabetic Peripheral Neuropathy Methods**

Datasources and Searching Methods

To identify studies in the published literature, we searched MEDLINE®, and the Cochrane Central Register of Controlled Trials (CENTRAL) from January 1, 2012 through May 25, 2016. We selected the January 2012 date restriction to overlap with the search dates of a relevant, high-quality systematic review.

We used a broad search to identify records in ClinicalTrials.gov. We used the advanced search function and entered the following terms: diabetic peripheral neuropathy [DISEASE] AND "Interventional" [STUDY-TYPES] AND NOT ("not yet recruiting" OR "terminated") [OVERALL-STATUS]. We ran the search on March 9, 2016. We downloaded all study fields for the search results as a comma-separated values file.

Study Selection and Matching with Peer-Reviewed Publications

Two reviewers independently assessed each ClinicalTrials.gov record for eligibility. We used the same eligibility criteria as the Diabetic Peripheral Neuropathy systematic review. We reviewed the ClinicalTrials.gov records using Microsoft Excel 2013 (Microsoft Corporation, Redmond, WA).

We matched ClinicalTrials.gov records to their published papers using their embedded PubMed citations and the National Library of Medicine’s National Clinical Trial Identifier (NCT) listed in published articles. Where we did not identify a match using the NCT identifier, we manually searched Medline using terms for the interventions and principal investigator as search criteria. Based on methods developed by Hartung and colleagues, we considered a PubMed publication to match a ClinicalTrials.gov registered trial if the intervention was the same AND 1 or more groups in the trial had an identical number of study participants. We used all publications that matched each trial.

Data Extraction

Two team members extracted data from ClinicalTrials.gov and matched publications. We extracted the following elements into pre-designed data extraction forms (Table 1) in Microsoft Excel 2013 (Microsoft Corporation, Redmond, WA). We developed two sets of evidence tables: the first set included only data from ClinicalTrials.gov, and the second set also had the data from the matched publications, if available.

Table 1. Data extraction elements

| Trial design | Design (parallel or crossover) |
| --- | --- |
|  | Number of groups |
|  | Trial start date, trial end date |
| Trial discontinuation | Early discontinuation?  Reason for discontinuation. |
| Ongoing trial | Any delays? Reasons for delays (if any) |
| Population | Total enrollment, sample size in each arm, drop-outs |
|  | Participants included in analysis for each outcome |
| Intervention and comparator | Description of the intervention and comparator |
| Outcomes | Description of pre-specified primary outcomes, number of primary outcomes |
|  | Description of secondary outcome |
| Analysis | Description of the pre-specified statistical analysis plan |
| Results of primary and secondary outcomes | Results, direction and magnitude, if any were reported |
| Adverse outcomes |  |
| Funding | Funding source and role |
| History of Changes | Summary of changes |

Assessment of Risk of Bias

We completed risk of bias assessment for any studies uniquely identified from ClinicalTrials.gov. We used the same tools as used for the published studies in our Diabetic Peripheral Neuropathy project (i.e., Cochrane Risk of Bias tool).

Data Synthesis

Question 1. Description of the Identified Studies

For the first question, we described all studies we identified in ClinicalTrials.gov. We reported *“Which studies were in the EPC report alone, ClinicalTrials.gov alone or in both?”* We described which studies are ongoing and which have been completed and trial completion dates (since it may take 1 year or longer for trial results to appear in peer reviewed literature).

Question 2. Comparison of Data Elements and Results from ClinicalTrials.gov and Matched Publications

Next, we addressed the second question, “*For the completed studies which were in both:*

*What were the differences, if any, in pre-specified outcome measures, statistical plan and size of the study reported in the peer reviewed literature vs. ClinicalTrials.gov?*

*Were results reported in ClinicalTrials.gov for any of the studies? If they were, what were the differences, if any, in the results reported in the peer reviewed literature vs. ClinicalTrials.gov*?”

Two reviewers compared the planned sample size, the primary outcome, and the analysis plan specified in the earliest version of the ClinicalTrials.gov record with what is reported in the corresponding publication. The earliest version of the ClinicalTrials.gov record was found under the History of Changes. Investigators independently assessed for discrepancies and then discussed these comparisons. Where discrepancies existed, we also reviewed the summary of changes from the ClinicalTrials.gov records to describe a rationale for the different results or plans.

We classified discrepancies between the elements extracted from ClinicalTrials.gov and the matched publications.

- **Identification of the primary outcome**. For assessing consistency of the pre-specified primary outcome (s), we used a framework developed by Zarin and colleagues.[^3^](#_ENREF_3) Applying this tool, the primary outcome could differ in the following ways: description of outcome (i.e. different “primary outcome” reported), different domain used, different measurement or diagnostic test used, different reporting of the same measure (e.g. change in pain scale or percentage from baseline), different results of the same reported measure. For trials with multiple publications and outcomes, we assessed each outcome separately, but designated one as the “main” primary.
- **Adverse event and deaths**. ClinicalTrials.gov began to mandate reporting of adverse events in September 2009 as serious adverse events and non-serious adverse events. We compared the total adverse events reported in ClinicalTrials.gov with the total reported in the matched publications.
- **Comparison of prespecified statistical plan**
- **Sample sizes, total.**

Question 3. Description of Incomplete or Discontinued Trials

We created separate tables for those studies that are incomplete or discontinued to address Question 3: *“For studies in ClinicalTrials.gov that were not completed or discontinued:*

*For the discontinued studies, were there reasons given for discontinuation? If so, what were they?*

*For studies that are ongoing but not completed, what was the date of initiation of the studies? Are the studies proceeding according to the original schedule or is there information in ClinicalTrials.gov indicating a delay in completion? If there is a delay in completion, what is the reason given?”*

These data were extracted as above to address this question.

Question 4. Incorporating the ClinicalTrials.gov Findings into the Review

The Diabetic Peripheral Neuropathy systematic review team graded the strength of evidence only for the outcomes identified as important and critical. They specified *a priori* that pain and quality of life were the most important and critical outcomes for assessing treatment options for symptoms of diabetic peripheral neuropathy. Therefore, we focus our assessment of the effects of searching ClinicalTrials.gov on these two outcomes.

We organized the results by comparison. For each outcome and comparator, we synthesized the body of evidence obtained with and without ClinicalTrials.gov. We highlighted discrepant outcomes and results between the published and unpublished results, based on our review, described above.

We conducted the following for each outcome by drug comparison:

- Describe the source of each study (published literature only, ClinicalTrials.gov only, or both)
  - For studies found in the published literature only, we noted those that were published prior to 2008, when Congress expanded the requirements of ClinicalTrials.gov.
  - For studies found in both the published literature and ClinicalTrials.gov, we compared the results for pain and quality of life that were reported in each source. We noted any additional or different outcomes and/or different or additional results.
  - For studies found in ClinicalTrials.gov only, we summarized the results for pain and quality of life.
- We qualitatively described the discordance (within an outcome and drug comparison) between results from ClinicalTrials.gov and published literature, in terms of direction of conclusions.
- Where ClinicalTrials.gov provided results, and we were able to conduct meta-analyses, we conducted sensitivity analyses, with and without the additional data from ClinicalTrials.gov.
- We considered if the final conclusions were influenced by any indication of reporting bias based on what was reported in ClinicalTrials.gov versus in the peer-reviewed literature.
- We graded the level evidence with and without the ClinicalTrials.gov results.

Throughout the process we logged challenges and issues, as well as tracked the time and effort to complete this work.

**Management of Infertility Methods**

**Scope and General Approach**

We adopted a pragmatic approach, using methods that could be readily incorporated into future systematic reviews. To maintain feasibility while still applying our methods to a range of interventions, we included Key Question (KQ) 1, KQ 2, and KQ 4 from the Management of Infertility SR in this analysis. The KQs are listed below:

**KQ 1:** What are the comparative safety and effectiveness of available treatment strategies for women with polycystic ovary syndrome (PCOS) who are subfertile/infertile and who wish to become pregnant?

**KQ 2:** What are the comparative safety and effectiveness of available treatment strategies for women with endometriosis who are subfertile/infertile and who wish to become pregnant?

**KQ 4:** What are the comparative safety and effectiveness of available treatments for women with tubal or peritoneal factors (e.g., pelvic adhesions) who are subfertile/infertile and who wish to become pregnant?

**Searching CT.gov**

We searched CT.gov for trials potentially applicable to the KQs with the assistance of our search librarian. Because CT.gov does not use MeSH-based search terms, we adapted the search strategies developed for the Management of Infertility SR to language appropriate for CT.gov. We conducted two searches—a broad search using the basic interface and a more specific search using the advanced interface in CT.gov. For the broad search, we searched for synonyms for infertility (infertility OR infertile OR subfertility OR subfertile OR sub-fertility OR sub-fertile) in the conditions field and limited our results to interventional studies. For the narrow search, we searched for the same synonyms for infertility in the broader search terms field and combined this with multiple, separate searches for each of the conditions of interest. This narrower search was also limited to interventional studies. Exact search strings used in both searches are given in Appendix A of the Management of Infertility SR.

Results of the two searches were imported into Excel.

**Matching Studies**

We matched randomized controlled trials (RCTs) identified in CT.gov with those identified for the Management of Infertility SR at several levels.

First, we determined whether RCTs reporting a live birth outcome that were included in the Management of Infertility SR had a matching record in CT.gov. Matching was performed initially using the NCT identifier (NCTID). Our intention was to conduct this matching using a semi-automated process within the bibliographical database (EndNote® Version X7; Thomson Reuters, Philadelphia, PA). This approach proved infeasible due to inconsistent assignment of NCTIDs to EndNote fields. Thus, all matching was accomplished by manual review. For unmatched studies, we conducted a secondary match using other trial registration numbers and then trial characteristics, including: condition, intervention, sample size, and author/investigator.

Matching was performed initially for the broad CT.gov search. We then determined the proportion of matched studies that were not identified by the narrow CT.gov search.

Second, for matched studies (i.e., studies included in the Management of Infertility SR with a CT.gov record), we abstracted selected variables from the CT.gov record to determine whether key study design variables and reported outcomes matched information in the published manuscript. Variables abstracted were:

• _Date of completion

• _Number of study arms

• _Intervention description

• _Study design

• _Outcomes measures and results prioritized in the Management of Infertility SR

• _Analysis approach

• _Subgroup analyses

Data from CT.gov were compared to published data. For each variable, the result was classified as: matching, discrepant, or possibly discrepant. Discrepant data were defined as cases where information was absent in one source but reported in another, or when the information given in the two sources was contradictory. Discrepancies were summarized narratively.

Third, we screened the unmatched CT.gov citations for potentially eligible completed trials. Eligibility criteria for each KQ are given in Table 1 of the Methods chapter of the main Management of Infertility SR. For potentially eligible studies identified from CT.gov, we used author names and intervention terms to search for a matching publication in PubMed. We classified studies into two groups: (1) potentially eligible completed study without a published manuscript; and (2) potentially eligible completed study with a matching published manuscript that was not identified in the systematic review search.

All matching was limited to studies published since the 2005 International Committee of Medical Journal Editors (ICMJE) policy requiring trial registration. Matching was performed initially by a research assistant, and reviewed by a study investigator. Team members involved in matching piloted the data collection forms and procedures to refine them before full use.

**Estimate of Person-Hours Required to Complete the Project**

EPC staff routinely log the time spent working on projects using project-specific codes. Co-investigators do not log project time routinely. Therefore, our project coordinator sent regular queries to co-investigators asking for estimates of time spent (to nearest 15 minutes) completing project-specific tasks. These estimates were tracked in an Excel spreadsheet. We used the staff logs and co-investigator reports to estimate the total staff time and co-investigator time dedicated to completing project-related activities.

**Impact on Systematic Review Conclusions**

Study conclusions will flow from the strength of evidence (SOE). We used the GRADE framework for evaluating SOE, a framework that includes assessment of risk of bias, consistency, precision, directness, and publication bias. The EPC risk of bias tool explicitly considers reporting bias. Therefore, risk of bias and publication bias are the domains most likely to be affected by supplemental data from CT.gov. In collaboration with authors of the Management of Infertility SR, we reviewed the SOE table to determine qualitatively whether study conclusions would change.

**Omega-3 Fatty Acids and Cardiovascular Disease Methods**

**Overview**

The Brown EPC conducted a review of the relationship between n-3 FA intake and CVD outcomes, following Institute of Medicine standards and Agency for Healthcare Research and Quality (AHRQ) guidance. This review (hereafter, “original review”) did not include registry searches as part of the strategy to identify ongoing studies.

We searched ClinicalTrials.gov and ICTRP up to the last search date of the original review (6/8/2015) to identify additional studies not identified in the original review, or additional information on the design or results of studies included in the original review.

**Terminology**

We use the term *study* to refer to the conducted research; a study may have one or more corresponding *registry records* in ClinicalTrials.gov or ICTRP *registries*, and these study results may be reported in the peer-reviewed literature as *publications*. A registry record provides basic information about a study’s design, and may include optional information on its results or publications associated with it. Studies identified through the registry search may have no associated publications; studies identified by the original report may have no records in a registry. A study was deemed to have been registered *prospectively* registration of data (defined here as registration of investigational studies prior to enrollment of the first patient or, for observational studies, prior to initial analyses.

**Registry Searches**

Because the registry databases are not indexed, queries can include only text words. Thus, it was necessary to translate the search of the original review, which includes text words, as well as controlled-vocabulary (MeSH) terms, to a semantically equivalent query using the registry interfaces. In addition, the ClinicalTrials.gov search interface allows only for queries with a limited number of characters, and documentation on advanced searching options, such as truncation and adjacency searching, is sparse. It is therefore better to search for “intervention” terms only. We conducted four queries in ClinicalTrials.gov whose union corresponded to the scope of the original search; we used an analogous search process in ICTRP.

**Databases:** ClinicalTrials.gov 8/14/2015 (5084 unique citations)

Search 1: Omega 3 OR Omega3 OR Omega-3 OR Fish OR n-3 OR Docosahexaenoic OR DHA OR Eicosapentaenoic OR EPA OR ALA OR alpha linolenic OR alphalinolenic OR alpha-linolenic OR fatty acids OR fatty acid OR PUFA OR SDA OR stearidonic

Search 2: Ropufa OR MaxEPA OR Omacor OR Efamed OR ResQ OR Epagis OR Almarin OR Coromega OR Lovaza OR Vascepa OR icosapent ethyl OR mediterranean diet

Search 3: salmon OR mackerel OR herring OR tuna OR halibut OR seaweed OR anchovy OR anchovies OR sardine OR sardines OR cod liver oil OR codliver oil OR marine oil

Search 4: walnut OR walnuts OR butternut OR butternuts OR soybean OR soybeans OR pumpkin seed OR pumpkin seeds OR flax OR flaxseed OR flax seed OR linseed OR rape seed OR rapeseed OR canola OR soy OR soybean OR walnut OR mustard seed OR perilla OR shiso

**Databases:** ICTRP 8/14/2015 (3468 unique citations)

Omega 3 OR Omega3 OR Omega-3 OR Fish OR n-3 OR Docosahexaenoic OR DHA OR Eicosapentaenoic OR EPA OR ALA OR alpha linolenic OR alphalinolenic OR alpha-linolenic OR fatty acids OR fatty acid OR PUFA OR SDA OR stearidonic OR Ropufa OR MaxEPA OR Omacor OR Efamed OR ResQ OR Epagis OR Almarin OR Coromega OR Lovaza OR Vascepa OR icosapent ethyl OR mediterranean diet OR salmon OR mackerel OR herring OR tuna OR halibut OR seaweed OR anchovy OR anchovies OR sardine OR sardines OR cod liver oil OR codliver oil OR marine oil OR walnut OR walnuts OR butternut OR butternuts OR soybean OR soybeans OR pumpkin seed OR pumpkin seeds OR flax OR flaxseed OR flax seed OR linseed OR rape seed OR rapeseed OR canola OR soy OR soybean OR walnut OR mustard seed OR perilla OR shiso

**Screening, Data Extraction, and Data Management**

Registry records were screened using the same approach employed in the original review (Appendix B). An evidence map comprised of registry records for eligible comparative and non-comparative studies was compiled, without minimum sample size or minimum follow up requirements. Basic study information (intervention, outcome, study design, sample size, and follow up duration) was recorded, noting if results were reported in the registry. Additional data was extracted from records that (1) included results and (2) met full eligibility criteria for the original report. These data include detailed study population data, the intervention details (i.e., n-3 FA type, dose, and duration), the reported outcomes, the numerical results, and on methodological items to assess the study risk of bias.

Data were extracted into the same customized forms developed and utilized for the original review in the Systematic Review Data Repository (SRDR) online system (http://srdr.ahrq.gov). Disagreements were resolved by discussion, with adjudication, when necessary, by the original report’s project lead.

**Analysis**

Our study yield was categorized as follows: 1) registry record present, included in original review; 2) registry record present, not included in original review; and 3) no registry record, included in original review. Characteristics of studies found exclusively in the original review, in a registry database, or in both sources, were documented.

Study initiation date, study status (e.g., discontinued, in progress/ongoing) and, when available, rationale for discontinuation or delay were also documented.

We quantified the number of studies and publications included in the original review but not found to have a registry record. We focused on the value of results data identified via registry searches, and thus in our analyses, we highlight the congruence, or lack thereof, among data identified via the registry and found in the original report in light of additional study data identified via registry searches.

Studies included in the original review and found to have a registry record were reviewed for additional information pertinent to study design (if the registry record includes protocol information) or study findings (if the record includes results). Study design information extracted from the registry record was compared to that extracted from corresponding publications to assess if changes in the outcomes or analysis plan occurred.

Comparisons between registry records and publications were made with respect to 1) general design items used to inform risk of bias assessments and 2) the analysis plan of the eligible exposure-outcome relationships. The risk of bias of each study result in the original review was evaluated based on predefined questions (Appendix C). We assessed whether additional information identified in registry records changed the risk of bias assessments of the original review.

When study results were identified in registry records and in corresponding publications, we determined if the same outcome concepts were employed, and if yes, whether the results agree qualitatively (i.e. same direction). We also describe which outcome measures were reported in the registry record, the publication, or both.

Registry records of newly identified studies (not included in the original review) are summarized in narrative form and added to the original report’s evidence map. We applied the same risk of bias assessments as in the original review (Appendix C).

**Risk of Bias for the Evidence Base and Strength of Evidence**

For outcomes with new data from the registries for specific n-3 FA comparisons, we reassessed the risk of bias of the evidence base and the strength of evidence using the same methodology used for the original report. We evaluated whether the additional data are likely to impact the findings of the study. We quantified this impact as a potential increase in total study population sample size (>20%), a change in the magnitude of outcome measures (20% change in estimate or a change in direction; ideally by meta-analysis), or a change in statistical significance (ideally by meta-analysis). Because meta-analyses were not conducted for most outcomes, we assessed whether the results from the new studies fall within the range of similar studies from the original report. If none of these conditions were met, the additional data were considered unlikely to directly impact the strength of evidence or the assessment of risk of bias for the evidence-base. All potential revisions to risk of bias and strength of evidence were discussed with the project lead of the original review. We describe and explain any changes to strength of evidence for any n-3 FA and outcome relationship.

**Tympanostomy Tubes in Children With Otitis Media Methods**

**Overview**

This report is based on a systematic review that is currently being conducted by our EPC on the relationship between tympanostomy tubes and a variety of outcomes, including hearing, developmental outcomes and quality of life, adverse events, and otorrhea. The ongoing systematic review (hereafter referred to as “original review”) is being conducted in accordance to IOM standards and AHRQ guidance.

**Terminology**

We use the term study to refer to the conducted research. Information about the design or results of studies may be reported in publications or in registry records. It is possible that studies identified through the registry search have no associated publications; and that studies identified in the original review have no records in ClinicalTrials.gov or ICTRP.

**Registry Searches**

Because the registry databases are not indexed, queries can only include text words. Thus, it was necessary to translate the search of the original review, which includes text words, as well as controlled-vocabulary (MeSH) terms, to a semantically equivalent query using the ClinicalTrials.gov and ICTRP interfaces. The ClinicalTrials.gov search interface allows only for queries with a limited number of characters, and documentation on advanced searching options, such as truncation and adjacency searching, is sparse. We were able to keep the PubMed search strategy intact for the ClinicalTrials.gov search, merely translating MeSH terms to text words. However, to run the search in the ICTRP interface, which does not allow for nested queries, we were forced to search only on intervention terms.

**ClinicalTrials.gov search 2/9/16**In interventions: tympanostomy OR grommet OR grommets OR tube OR tubes OR ventilation OR t-tube OR tubulation OR otologic surgical procedures
In Conditions: otitis OR "glue ear" OR middle ear OR OME OR SOM OR AOM
Limit to child
71 records retrieved
Searching the basic interface with the same terms:
(tympanostomy OR grommet OR grommets OR tube OR tubes OR ventilation OR t-tube OR tubulation OR otologic surgical procedures) AND (otitis OR "glue ear" OR middle ear OR OME OR SOM OR AOM)
Not limited to adults.
133 records retrieved
36 were labeled adult or adult/senior
26 remaining non-overlapping

**WHO ICTRP search 2/9/16**(otitis OR "glue ear" OR middle ear OR OME OR SOM OR AOM)

retrieved 586 records

**Analysis**

Registry searches were categorized as follows (1) included in the original review but not found in the registry, (2) included in the original review and found in a registry but with no new results data, (3) included in the original review and found in a registry with new data, and (4) identified via the registry but not found in the original review. Though we planned to document rationale for study discontinuation, none were provided for studies included in our analysis. We focus on the value of results data identified via registry searches, and thus highlight the congruence, or lack thereof, among data identified via the registry and found in the original review in light of additional study data identified via registry searches.

For studies included in the original review that also have a registry record, the additional information in the registry records pertains to their design (if the registry record includes protocol information) or their findings (if the record includes results). Information found in records was examined against information obtained from publications to judge whether important changes in the analysis plan occurred. We made such comparisons only with respect to 1) general design items used to inform risk of bias assessments and 2) the analysis plan of the eligible exposure-outcome relationships. The risk of bias of each study result in the original review will be evaluated based on predefined questions. We assessed whether the additional information in the registry records changed the risk of bias assessments in the original review. In the assessment for changes in the analysis plan, we looked for changes in the population studied, the effect measure (e.g., difference in means, odds ratios for specific categorizations of continuous outcomes), and maximum follow up recorded; we also looked for differences in the estimation procedure (the prescribed statistical learning procedure) and the plan for handling missing values, where the records give sufficient information.

We have described whether registry records and publications describe the same outcomes. Because no records with matched publications gave results, we were unable to assess whether the results agreed. Registry records of newly identified studies not included in the original review are summarized in narrative form and extracted into spreadsheets based on the original review’s extraction form. We applied the same risk of bias assessments as in the original review, where it is possible to assess risk of bias, and report results, as well as whether the new results can be incorporated into the analyses of the original review.

**Risk of Bias for the Evidence Base and Strength of Evidence**

For outcomes with new data from the registries, we have reassessed the risk of bias of the evidence base and the strength of evidence using the same methodology used for the original review. We assess whether the new studies fall within the range of the similar studies from the original review and whether the additional data are likely to directly impact the strength of evidence or the assessment of risk of bias for the evidence-base.

**Strategies To Improve Mental Health Care for Children and Adolescents Methods**

**KQ 1**

We updated our searches for SIMHC draft report and then compare the yield with clinicaltrials.gov, using a dual independent review process.

**KQ 2**

(a) For studies with information in both peer-reviewed literature and clinicaltrials.gov, we extracted and compared the results, using a dual review process, with a second reviewer checking the first abstractions.

(b) For studies with differences in reporting by source, we reached out to study authors via email and phone interview, if necessary, to understand the reasons for the differences.

**KQ 3**

(a) For discontinued studies, we planned to reach out to authors via email to identify reasons for discontinuation.

(b) For ongoing incomplete studies, we supplemented information in clinicaltrials.gov with additional information from study authors via email.

(c) For completed and unpublished studies, we planned to reach out to authors of discontinued studies via email to identify reasons for lack of publication

**KQ 4**

We reached out to authors of included studies on the reasons for use or non-use of clinicaltrials.gov or other archive sites for information on study conduct and processes.

**KQ 5**

We integrated the information for KQs 1-4, using data from searches; abstraction from clinicaltrials.gov; and email, personal interviews, and any additional information provided by authors. We planned to update the strength of evidence and conclusion of the SIMHC report, if we found relevant results.

Table 1 provides the questions for email or personal interview. These are general questions, to be tailored for each interviewee. We obtained IRB exemption before conducting email interviews. We planned a minimum of two email and two telephone outreach attempts before categorizing investigators as non-responders.

**Table 1. Questions for authors of studies identified for the SIMHC report or through clinicaltrials.gov**

| The RTI-UNC Evidence-based Center is conducting a systematic review of strategies to improve mental health for children and adolescents. In addition, our funder, the Agency for Healthcare Research and Quality, has requested an additional investigation of the validity and reliability of clinicaltrials.gov as a potential additional source of information on study conduct, processes and results. Your study [xxx, has been included/is eligible for inclusion] in this review. We are reaching out to you to obtain some additional details about the reporting of your study. Thank you for agreeing to answer our questions. |
| --- |
| [For authors of included clinical trials included in the report that do not have a clinicaltrials.gov listing, N=8]   1. We were unable to find a listing for your study on clinicaltrials.gov. Is the study listed on clinicaltrials.gov? If yes, what is the listing number? 2. Is the study listed elsewhere on another clinical trials registry? If yes, where and what is the listing number? 3. [If the study results are not listed in any clinical trials registry] Did you attempt to list your study in a clinical trials registry? If yes, what barriers did you experience? 4. Where can other investigators find supplemental information on your study, such as your experiences with implementing the study or your assessment of critical components necessary for dissemination? 5. In abstracting your study, we noted that study arms differed in their use of [list specific components here, tailored for each study]. Which of these elements (or otherwise that we may be unaware of) do you consider to be the critical component(s) of your intervention, for those wishing to replicate your study? |
| [For authors of clinical trials included in the report that have a listing in clinicaltrials.gov, with no results reported in clinicaltrials.gov at the time of our outreach, N=5]   1. What barriers did you experience or anticipate in presenting your results in a clinical trials registry? 2. If other investigators wish to scale up your strategy, where can they find necessary information, for example, on your experience of study conduct and processes or your assessment of critical components? 3. What do you consider to be the critical components of your intervention, for those wishing to replicate your study? 4. [If such information is not available publicly or in clinicaltrials.gov] What barriers did you experience or anticipate in using a clinical trials registry to make such information available publicly? |
| [For authors of studies included in the report that are NOT clinical trials, N=4]   1. If other investigators wish to scale up your strategy, where can they find information on your experience of study conduct and processes or your assessment of critical components necessary for dissemination? 2. In abstracting your study, we noted that study arms differed in their use of [list specific components here, tailored for each study]. Which of these elements (or otherwise that we may be unaware of) do you consider to be the critical component(s) of your intervention, for those wishing to replicate your study? 3. [If such information is not available publicly] Are you aware of public registries for observational or non-randomized studies that might be relevant to your effort? If yes, what are these registries? 4. What barriers did you experience or anticipate in using registries to make information on study conduct and processes available publicly? |
| [For authors of ongoing incomplete studies identified via clinicaltrials.gov, not included in the SIMHC review, N=3]   1. We identified your ongoing study through a search of clinicaltrials.gov as potentially meeting our eligibility criteria for inclusion in our SIMHC review. [If clinicaltrials.gov does not provide this information] What is the anticipated date of completion for this study? 2. We identified your study through a search of clinicaltrials.gov as potentially meeting our eligibility criteria for inclusion in our SIMHC review. Are there plans to publish the findings? If yes, where will you attempt to publish the material? If no, why not? 3. Is there any addition information or data that you could share with us that is not currently included on clincaltrials.gov for this study? [If relevant] 4. Your experience of study conduct and processes may be valuable to others attempting a similar strategy. Where can other investigators find such information? 5. [If relevant] What barriers did you experience or anticipate in using a clinical trials registry to make such information available publicly? |

We also constructed questionnaires in three additional categories but did not find studies in these categories (studies with different results reported in clinicaltrials.gov and published results, eligible discontinued studies identified via clinicaltrials.gov, and complete but unpublished studies identified via clinicaltrials.gov)
